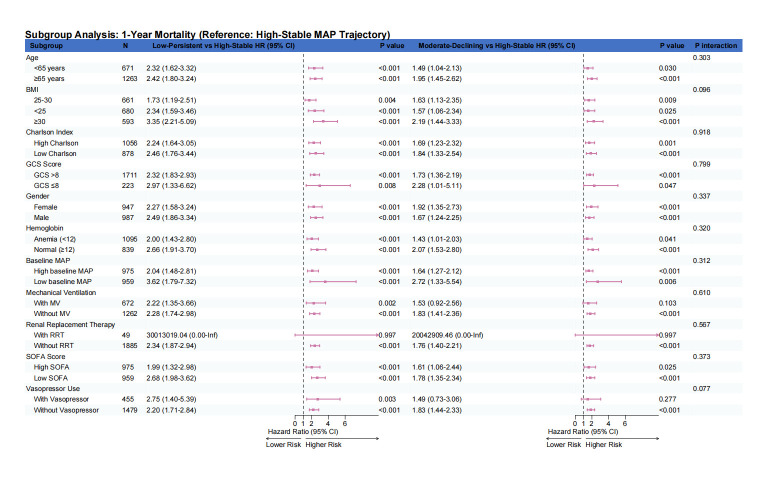# Association of early mean arterial pressure trajectory subtypes with mortality in critically ill patients with ischemic stroke: A multicenter retrospective cohort study

**DOI:** 10.1097/MD.0000000000048999

**Published:** 2026-05-29

**Authors:** Jintao Qiu, Rongrong Liu

**Affiliations:** a Shenzhen Yantian District People’s Hospital, Shenzhen, Guangdong, China.

**Keywords:** critical care, ischemic stroke, mean arterial pressure, mortality, trajectory analysis

## Abstract

Mean arterial pressure (MAP) management is crucial for cerebral perfusion in critically ill patients with ischemic stroke. However, prior studies have predominantly focused on static blood pressure measurements, and the prognostic relevance of early dynamic MAP patterns after intensive care unit (ICU) admission remains insufficiently characterized. This study aimed to identify distinct MAP trajectory subtypes during the first 24 hours after ICU admission and evaluate their associations with mortality in critically ill patients with ischemic stroke. This multicenter retrospective cohort study used data from Medical Information Mart for Intensive Care IV (MIMIC-IV) v3.1 (development cohort) and eICU v2.0 (external validation cohort). Adult patients with ischemic stroke and complete hourly MAP data during the first 24 hours of ICU admission were included. Group-based trajectory modeling identified distinct MAP trajectory subtypes. Cox proportional hazards regression was used to assess associations with in-hospital mortality in both cohorts and with 28-day and 1-year mortality in MIMIC-IV. Exploratory effect-modification analyses included continuous interaction models for major continuous covariates, complemented by categorized subgroup displays for descriptive presentation. A total of 4734 patients were analyzed (MIMIC-IV: 1934; eICU: 2800). Three trajectory subtypes were identified: High-Stable (approximately 103–108 mm Hg), Moderate-Declining (93–87 mm Hg), and Low-Persistent (80–73 mm Hg). In MIMIC-IV, in-hospital mortality rates were 11.9%, 18.9%, and 23.6% for the High-Stable, Moderate-Declining, and Low-Persistent subtypes, respectively. Compared with the High-Stable subtype, the Low-Persistent subtype was associated with increased in-hospital mortality (HR: 2.03, 95% CI: 1.47–2.79), as was the moderate-declining subtype (HR: 1.62, 95% CI: 1.17–2.23). Similar associations were observed in the eICU cohort (low-persistent HR: 2.01, 95% CI: 1.51–2.69; moderate-declining HR: 1.61, 95% CI: 1.21–2.14) and across 28-day and 1-year mortality in MIMIC-IV. Continuous interaction analyses showed broadly similar associations across the observed range of major continuous covariates, and categorized subgroup displays yielded consistent descriptive patterns. Early MAP trajectory subtypes were associated with mortality in critically ill patients with ischemic stroke, with the low-persistent pattern showing the highest risk. These findings suggest that early MAP trajectories may reflect clinically relevant hemodynamic phenotypes; however, their incremental value beyond conventional static MAP measures was not directly assessed in this study.

## 1. Introduction

Acute ischemic stroke (AIS) remains a leading cause of death and long-term disability worldwide, and the global burden has continued to increase, underscoring the need for optimized acute management strategies across care settings.^[1–4]^ A substantial subset of AIS patients requires intensive care unit (ICU) admission for airway protection, close neurologic monitoring, advanced hemodynamic support, and management of early complications, where physiologic derangements may amplify secondary brain injury.

Blood pressure (BP) disturbances are common during the hyperacute and acute phases of AIS and are clinically consequential because cerebral perfusion may become pressure-dependent in the setting of impaired autoregulation and vulnerable penumbral tissue.^[5,6]^ Current guideline recommendationsin AIS provide clear thresholds in selected contexts (e.g., thrombolysis- and thrombectomy-related BP limits), but they are generally conservative and reflect persistent uncertainty regarding optimal hemodynamic targets for heterogeneous patient populations.^[7,8]^ In parallel, general critical care practice often uses mean arterial pressure (MAP) targets (commonly ≥ 65 mm Hg) as a foundational resuscitation goal for organ perfusion in shock states, yet the applicability of a “one-size-fits-all” MAP threshold to neurocritical AIS (where cerebral perfusion pressure and collateral flow are pivotal) remains insufficiently defined.^[9]^

Evidence from reperfusion-treated AIS populations further illustrates the complexity of BP management and the potential harm of overly aggressive BP reduction. In patients undergoing successful endovascular thrombectomy (EVT), randomized trials have reported that intensive BP lowering strategies may be neutral or associated with worse functional outcomes compared with more conventional targets, suggesting that excessive BP reduction after reperfusion can be detrimental.^[10–12]^ Contemporary meta-analyses similarly indicate that intensive post-EVT BP control does not confer clear benefit, may carry safety trade-offs, and therefore support a more conservative strategy while awaiting further trial evidence.^[6]^

Beyond absolute BP levels, increasing attention has shifted toward early BP dynamics, including variability and temporal patterns, as potentially more informative markers of physiological instability and therapeutic exposure. Beat-to-beat BP variability within the first 24 hours of AIS onset has been independently associated with worse short-term functional prognosis, highlighting the prognostic relevance of early BP fluctuations.^[13]^ In EVT cohorts, higher BP variability within the first 24 hours has been linked to increased mortality and disability independent of mean BP, suggesting that variability represents a distinct risk domain.^[14]^ Importantly, recent work by Zhou et al further reinforced this concept, reporting that increased BP variability during the first 24 hours after thrombectomy in basilar artery occlusion was associated with worse outcomes, particularly among patients without prior hypertension.^[15]^ Peri-procedural hypotension burden may also be harmful; a 2024 study reported that the magnitude of intra-procedural arterial hypotension during thrombectomy under general anesthesia was independently associated with unfavorable 90-day outcomes.^[16]^ Meanwhile, trajectory-based approaches are emerging as a clinically interpretable framework to capture dynamic BP evolution; distinct post-EVT BP trajectory groups have been shown to correlate with radiographic and functional outcomes, supporting the clinical interpretability of pattern-based phenotyping alongside single-point measurements.^[17]^

However, despite growing interest in BP variability and trajectories, evidence specifically centered on critically ill AIS patients after ICU admission, where vasoactive therapies, sedation, ventilation, and systemic organ dysfunction may substantially shape MAP exposure, remains limited, particularly regarding the prognostic impact of early (first 24 hours) MAP trajectory phenotypes. Therefore, in this study, we leveraged 2 large, multicenter critical care databases to identify distinct MAP trajectory subphenotypes during the first 24 hours after ICU admission in AIS patients and evaluate their associations with short- and longer-term mortality, with the goal of characterizing early MAP trajectory phenotypes rather than establishing direct bedside superiority over conventional static MAP assessment.

## 2. Materials and methods

### 2.1. Study design and data sources

This retrospective multicenter cohort study utilized data from 2 large publicly available critical care databases: the Medical Information Mart for Intensive Care IV (MIMIC-IV) version 3.1 (MIMIC-IV v3.1, released October 11, 2024; https://physionet.org/content/mimiciv/3.1/;^[18]^ and the eICU Collaborative Research). Database version 2.0 (eICU v2.0, released April 2019; https://physionet.org/content/eicu-crd/2.0/; PMID: 30204154). MIMIC-IV contains de-identified electronic health records from over 360,000 patients admitted to Beth Israel Deaconess Medical Center in Boston between 2008 and 2022, encompassing approximately 546,000 hospitalizations and 94,000 ICU admissions. The eICU database comprises de-identified health data from >200,000 ICU admissions across over 200 hospitals in the United States during 2014 to 2015, collected through the Philips-eICU telehealth program.^[19]^ MIMIC-IV served as the primary cohort for model development, while eICU was used for external validation. The collection of patient information and creation of the MIMIC-IV research resource was reviewed by the Institutional Review Board at Beth Israel Deaconess Medical Center, who granted a waiver of informed consent and approved the data sharing initiative. This study was conducted in accordance with the principles of the Declaration of Helsinki and adhered to the Strengthening the Reporting of Observational Studies in Epidemiology (STROBE) guidelines.

### 2.2. Study population

Adult patients (age ≥ 18 years) diagnosed with ischemic stroke were identified from both databases using International Classification of Diseases codes (ICD-9: 433.x1, 434.x1, 436; ICD-10: I63.x). The inclusion criteria required patients to have their first hospital admission with a single ICU stay and an ICU length of stay of at least 24 hours. For MAP trajectory analysis, hourly MAP values were calculated by averaging multiple measurements recorded within the same hour, and only patients with complete MAP data at all 25 time points (hours 0–24) were included. Patients with missing MAP values at any time point during this observation window were excluded. From MIMIC-IV, 3777 ischemic stroke patients were initially identified; 956 were excluded for non-first hospital admissions or multiple ICU admissions, 1 for age ≤18 years, 266 for ICU length of stay <24 hours, and 620 for incomplete hourly MAP data, yielding a final cohort of 1934 patients. From eICU, 4862 patients were initially identified; 1070 were excluded for non-first or multiple admissions, 1 for age criteria, 728 for insufficient ICU stay duration, and 263 for incomplete MAP measurements, resulting in 2800 patients for external validation.

### 2.3. Data extraction and variable definitions

Baseline characteristics were extracted at ICU admission (hour 0) and included demographic variables (age, gender, weight, and body mass index [BMI]), disease severity scores (Simplified Acute Physiology Score II, Oxford Acute Severity of Illness Score [OASIS], Sequential Organ Failure Assessment, Glasgow Coma Scale (GCS), and Charlson comorbidity index), vital signs (systolic and diastolic blood pressure, heart rate, respiratory rate, temperature, and peripheral oxygen saturation), laboratory parameters (white blood cell count, hemoglobin, platelet count, creatinine, blood urea nitrogen, blood glucose, sodium, and potassium), and clinical interventions within the first 24 hours (mechanical ventilation, renal replacement therapy [RRT], vasopressor use, insulin administration, and sedative use). MAP values were recorded at hourly intervals from ICU admission through 24 hours for trajectory analysis. The primary outcome was in-hospital mortality. Secondary outcomes included 28-day mortality and 1-year mortality, which were available only in the MIMIC-IV cohort.

### 2.4. Group-based trajectory modeling

Group-based trajectory modeling (GBTM) was employed to identify distinct MAP trajectory patterns during the first 24 hours of ICU admission. GBTM is a specialized application of finite mixture modeling that identifies clusters of individuals following similar developmental trajectories over time. Models with 2 to 5 trajectory groups were fitted, and the optimal number of groups was determined based on the Bayesian Information Criterion, average posterior probability of group membership (≥0.70), and clinical interpretability. Each patient was assigned to the trajectory group with the highest posterior probability. Three distinct MAP trajectory subtypes were identified and labeled according to their hemodynamic characteristics: High-Stable (characterized by consistently elevated MAP levels), Moderate-Declining (intermediate MAP with declining trend), and Low-Persistent (persistently low MAP throughout the observation period).

### 2.5. External validation

The GBTM-derived trajectory classification model developed in MIMIC-IV was externally validated in the eICU cohort. Patients in the validation cohort were assigned to trajectory subtypes based on minimum Euclidean distance to the centroid trajectories established from the training cohort. The consistency of trajectory patterns and their prognostic associations were evaluated across both databases.

### 2.6. Statistical analysis

Baseline characteristics were compared across databases using standardized mean differences. Continuous variables were presented as median with interquartile range due to non-normal distributions assessed by Shapiro–Wilk tests, while categorical variables were expressed as frequencies and percentages. MAP temporal trends were visualized by calculating mean values with 95% confidence intervals at each time point, stratified by survival outcome. Kaplan–Meier survival curves were constructed to compare mortality outcomes among trajectory subtypes, with log-rank tests for statistical comparison. Cox proportional hazards regression was performed to estimate hazard ratios with 95% confidence intervals, using the High-Stable subtype as the reference group given its most favorable hemodynamic profile. Exploratory effect-modification analyses were performed to assess whether the association between MAP trajectory subtypes and mortality varied across selected clinical variables. For major continuous variables, including age, BMI, GCS score, hemoglobin, baseline MAP, sequential organ failure assessment (SOFA) score, and Charlson comorbidity index, we fitted Cox proportional hazards models including trajectory group, the continuous covariate, and their interaction term, and estimated hazard ratios across the observed range of each covariate. These continuous interaction analyses were used as the primary approach to evaluate potential effect modification while preserving the continuous information in the data. In addition, for descriptive presentation and ease of clinical interpretation, we retained categorized exploratory subgroup analyses shown as forest plots. In these display-oriented analyses, age was categorized as <65 or ≥65 years, BMI as <25, 25 to 30, or ≥30 kg/m^2^, GCS as >8 or ≤8, hemoglobin as <12 or ≥12 g/dL, and mechanical ventilation, vasopressor use, and RRT as yes/no; baseline MAP, SOFA score, and Charlson comorbidity index were dichotomized using distribution-based thresholds. These categorized analyses were intended to complement, rather than replace, the continuous interaction analyses. Heatmaps were generated to visualize baseline clinical feature distributions across trajectory subtypes, with continuous variables standardized as *z*-scores and compared using Kruskal–Wallis tests, while categorical variables were compared using chi-square or Fisher exact tests as appropriate. All statistical analyses were performed using R version 4.3.0 with packages including tableone, survival, survminer, lcmm, and ComplexHeatmap. A two-sided *P* value <.05 was considered statistically significant.

## 3. Results

### 3.1. Baseline characteristics

**Figure 1.**
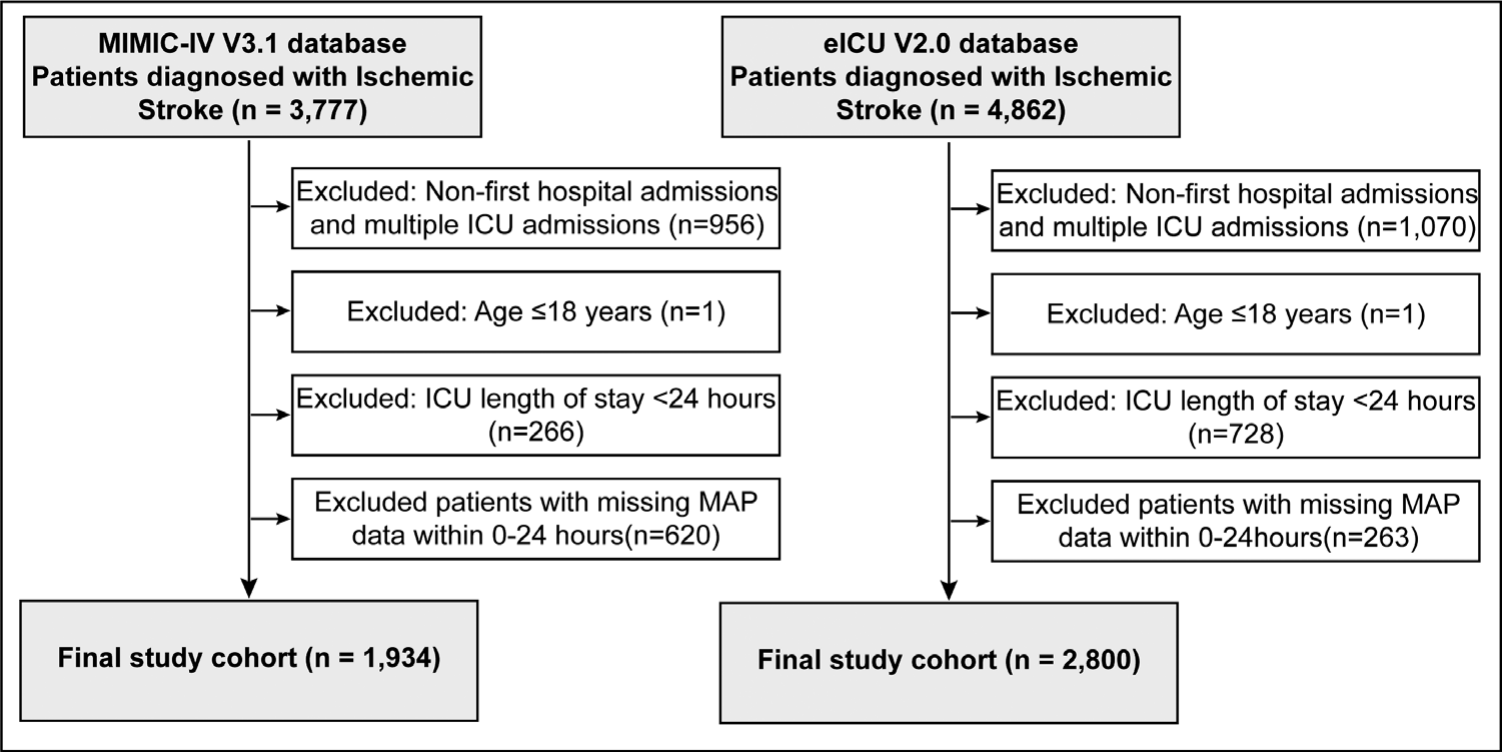
Flowchart of patient selection and exclusion criteria. Patients diagnosed with ischemic stroke were identified from the MIMIC-IV v3.1 database (n = 3777) and eICU v2.0 database (n = 4862). After applying exclusion criteria for non-first hospital admissions, multiple ICU admissions, age ≤18 years, ICU length of stay <24 hours, and incomplete hourly MAP data across the first 24 hours, the final study cohorts comprised 1934 patients from MIMIC-IV (development cohort) and 2800 patients from eICU (external validation cohort). ICU = intensive care unit, MAP = mean arterial pressure, MIMIC-IV = Medical Information Mart for Intensive Care IV.

A total of 8639 patients with ischemic stroke were initially identified from the MIMIC-IV (n = 3777) and eICU (n = 4862) databases. After application of the predefined exclusion criteria, 1934 patients from MIMIC-IV and 2800 patients from eICU were included in the final analysis (Fig. 1). Because trajectory modeling required complete hourly MAP values across the first 24 hours after ICU admission, patients with incomplete MAP measurements during this window were excluded. We therefore additionally compared otherwise eligible patients with complete versus incomplete early MAP data (Table S2, Supplemental Digital Content). This comparison suggested the possibility of selection bias related to data completeness, particularly in the eICU cohort. Missingness for the remaining baseline variables was generally low in both databases, with most variables showing <10% missing data (Table S1, Supplemental Digital Content).

The baseline characteristics of the study population stratified by database are summarized in Table 1. The median age was 71.00 years (IQR: 60.00–81.00) in MIMIC-IV and 70.00 years (IQR: 59.00–79.00) in eICU, with comparable gender distributions (male: 51.0% vs 51.4%). Patients in MIMIC-IV demonstrated higher disease severity as indicated by elevated Simplified Acute Physiology Score II scores (34.00 vs 28.00), OASISs (32.00 vs 26.00), and Charlson comorbidity index (7.00 vs 4.00; standardized mean difference = 1.090). MIMIC-IV patients also exhibited greater utilization of vasopressors (23.5% vs 11.4%) and sedatives (32.5% vs 23.2%). Regarding laboratory parameters, MIMIC-IV patients showed lower hemoglobin levels (11.50 vs 12.20 g/dL) and higher potassium concentrations (4.10 vs 3.90 mmol/L). The in-hospital mortality rate was significantly higher in MIMIC-IV compared to eICU (19.1% vs 12.7%, *P* < .001).

**Table 1:** Baseline characteristics of ischemic stroke patients stratified by database.

Characteristics	Level	MIMIC-IV (n = 1934)	eICU (n = 2800)	*P* value	SMD
Age (yr)		71.00 [60.00, 81.00]	70.00 [59.00, 79.00]	.025	0.055
Gender	Male	987 (51.0)	1438 (51.4)	.850	0.006
	Female	947 (49.0)	1362 (48.6)		
Weight (kg)		76.32 [64.58, 91.00]	79.50 [67.30, 94.50]	<.001	0.144
Body mass index (kg/m^2^)		27.14 [23.71, 30.99]	27.59 [23.95, 31.97]	.002	0.092
SAPS II score		34.00 [26.00, 44.00]	28.00 [21.00, 38.00]	<.001	0.421
OASIS score		32.00 [26.00, 38.00]	26.00 [21.00, 34.00]	<.001	0.506
SOFA score		4.00 [2.00, 6.00]	5.00 [3.00, 7.00]	<.001	0.199
Glasgow Coma Scale		14.00 [11.00, 15.00]	13.00 [9.00, 14.60]	<.001	0.341
Charlson comorbidity index		7.00 [5.00, 9.00]	4.00 [2.00, 5.00]	<.001	1.090
Systolic blood pressure (mm Hg)		134.00 [117.00, 151.75]	142.00 [123.75, 160.00]	<.001	0.287
Diastolic blood pressure (mm Hg)		76.00 [63.00, 88.00]	75.00 [63.00, 88.00]	.935	0.006
Heart rate (bpm)		82.00 [70.00, 96.00]	80.00 [69.00, 95.00]	.002	0.085
Respiratory rate (breaths/min)		19.00 [16.00, 23.00]	18.00 [16.00, 22.00]	.071	0.043
Temperature (°C)		36.78 [36.50, 37.06]	36.72 [36.40, 37.00]	<.001	0.103
SpO_2_ (%)		98.00 [96.00, 100.00]	98.00 [96.00, 100.00]	.434	0.057
White blood cell (×10^9^/L)		10.40 [7.90, 14.10]	9.85 [7.90, 12.60]	<.001	0.173
Hemoglobin (g/dL)		11.50 [9.60, 13.20]	12.20 [10.80, 13.50]	<.001	0.321
Platelet (×10^9^/L)		204.00 [155.25, 262.00]	208.00 [169.55, 254.00]	.030	0.042
Creatinine (mg/dL)		1.00 [0.80, 1.30]	0.90 [0.73, 1.20]	<.001	0.168
Blood urea nitrogen (mg/dL)		18.00 [13.00, 28.00]	17.00 [12.00, 24.00]	<.001	0.192
Blood glucose (mg/dL)		129.50 [104.00, 172.00]	125.00 [103.00, 159.00]	.002	0.123
Sodium (mmol/L)		139.00 [136.00, 141.00]	139.00 [137.00, 141.00]	.001	0.098
Potassium (mmol/L)		4.10 [3.80, 4.50]	3.90 [3.60, 4.20]	<.001	0.353
Mechanical ventilation	No	1262 (65.3)	1920 (68.6)	.018	0.071
	Yes	672 (34.7)	880 (31.4)		
Renal replacement therapy	No	1885 (97.5)	2753 (98.3)	.052	0.060
	Yes	49 (2.5)	47 (1.7)		
Vasopressor use	No	1479 (76.5)	2480 (88.6)	<.001	0.323
	Yes	455 (23.5)	320 (11.4)		
Insulin use	No	1507 (77.9)	2243 (80.1)	.074	0.054
	Yes	427 (22.1)	557 (19.9)		
Sedative use	No	1306 (67.5)	2149 (76.8)	<.001	0.207
	Yes	628 (32.5)	651 (23.2)		
In-hospital mortality	Survived	1565 (80.9)	2445 (87.3)	<.001	0.176
	Died	369 (19.1)	355 (12.7)		

Notes: Continuous variables are presented as median [IQR]. Categorical variables are presented as n (%). MIMIC-IV was used for model development; eICU was used for external validation.

GCS = Glasgow Coma Scale, ICU = intensive care unit, MIMIC-IV = Medical Information Mart for Intensive Care IV, OASIS = Oxford Acute Severity of Illness Score, SMD = standardized mean difference, SOFA = sequential organ failure assessment, SpO_2_ = peripheral oxygen saturation.

### 3.2. MAP trajectories stratified by survival outcome

**Figure 2.**
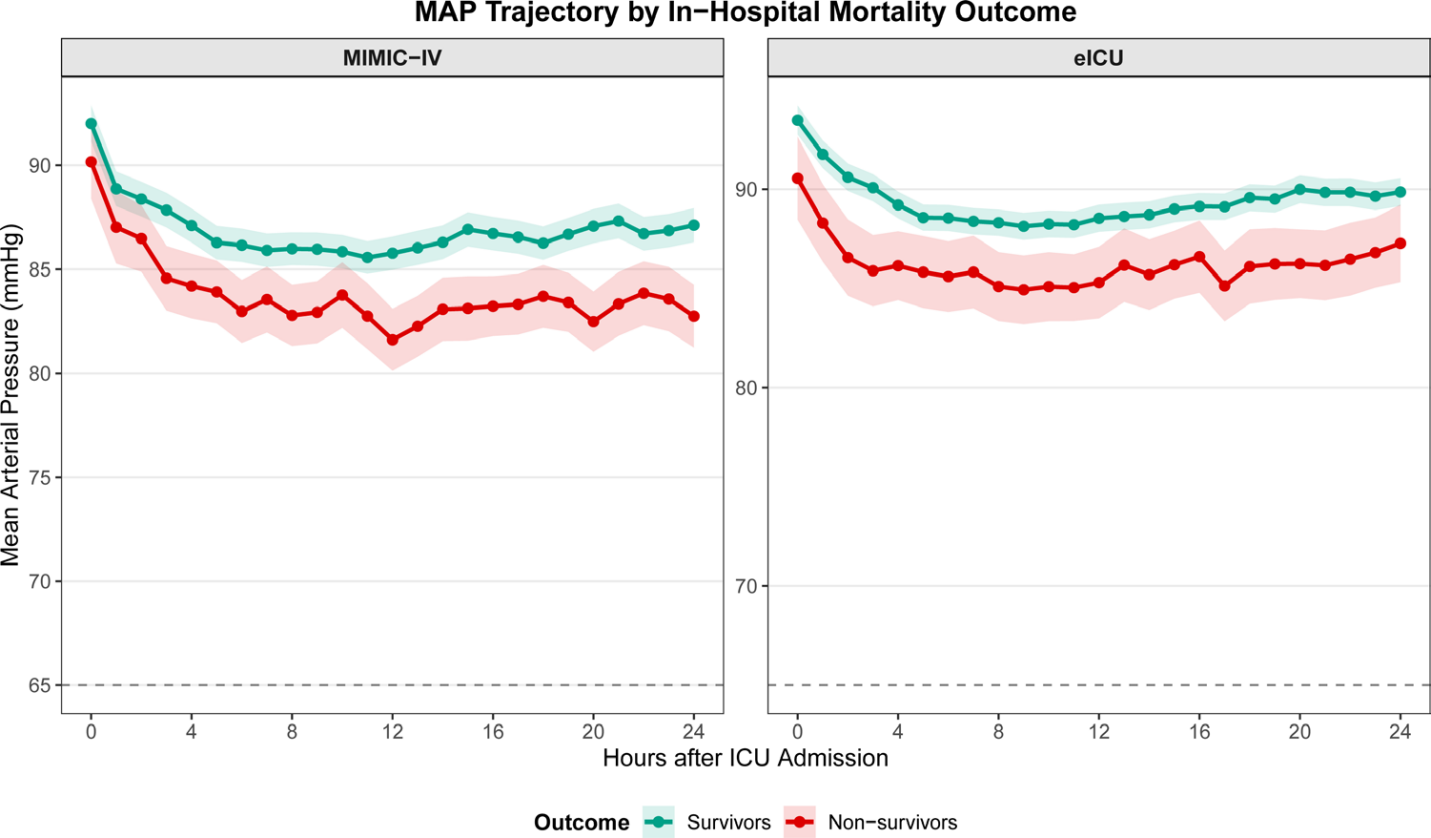
Mean arterial pressure trajectories during the first 24 hours of ICU admission stratified by in-hospital mortality outcome. Lines represent mean MAP values and shaded areas indicate 95% confidence intervals. The horizontal dashed line indicates the MAP threshold of 65 mm Hg. (A) MIMIC-IV cohort; (B) eICU cohort. ICU = intensive care unit, MAP = mean arterial pressure, MIMIC-IV = Medical Information Mart for Intensive Care IV.

To further characterize the hemodynamic profiles associated with clinical outcomes, MAP trajectories during the first 24 hours of ICU admission were analyzed according to in-hospital mortality status (Fig. 2; Table S3, Supplemental Digital Content). In the MIMIC-IV cohort (Fig. 2A), survivors demonstrated consistently higher MAP values compared to non-survivors throughout the observation period. At ICU admission (hour 0), survivors had a mean MAP of 92.0 ± 17.3 mm Hg compared to 90.2 ± 17.2 mm Hg in non-survivors. This difference became more pronounced over time, with survivors maintaining a mean MAP of 87.1 ± 16.5 mm Hg at hour 24, whereas non-survivors exhibited a lower value of 82.7 ± 14.8 mm Hg. The trajectory pattern in non-survivors was characterized by an initial decline from admission, reaching a nadir around hour 12 (81.6 ± 14.6 mm Hg), followed by partial recovery but persistently lower levels than survivors.

Similar patterns were observed in the eICU validation cohort (Fig. 2B). Survivors maintained higher MAP values throughout, with baseline MAP of 93.5 ± 18.4 mm Hg decreasing to a stable plateau around 88 to 90 mm Hg. Non-survivors showed lower initial MAP (90.5 ± 20.1 mm Hg at hour 0) with a declining trend to approximately 85 to 87 mm Hg during hours 8 to 12, followed by modest recovery to 87.3 ± 18.7 mm Hg at hour 24. Notably, both cohorts demonstrated that the MAP difference between survivors and non-survivors was most evident during the middle phase of the observation window (hours 6–14), suggesting this period may be critical for hemodynamic stabilization in ischemic stroke patients.

### 3.3. Identification of MAP trajectory subtypes

**Figure 3.**
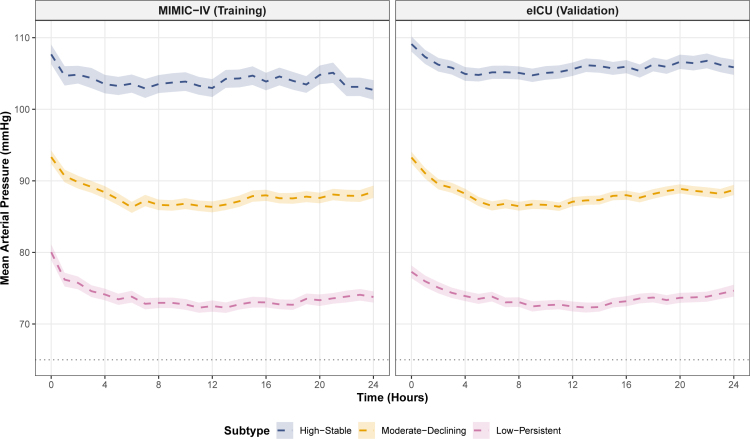
MAP trajectory subtypes identified by group-based trajectory modeling among ischemic stroke patients. Three distinct trajectory patterns were identified: High-Stable, Moderate-Declining, and Low-Persistent. Solid lines indicate mean MAP values, and shaded areas indicate 95% confidence intervals. The horizontal dashed line indicates the MAP threshold of 65 mm Hg. (A) MIMIC-IV training cohort; (B) eICU validation cohort. ICU = intensive care unit, MAP = mean arterial pressure, MIMIC-IV = Medical Information Mart for Intensive Care IV.

Given the observed differences in MAP trajectories between survivors and non-survivors, group-based trajectory modeling was applied to identify distinct hemodynamic patterns during the first 24 hours of ICU admission. The optimal model identified 3 trajectory subtypes with good clustering performance (Silhouette index: 0.408; Calinski-Harabasz index: 885; Davies-Bouldin index: 1.487) (Fig. 3). In the MIMIC-IV training cohort, 412 patients (21.3%) were classified as High-Stable, characterized by consistently elevated MAP values (107.7 ± 14.0 mm Hg at baseline, maintaining approximately 103–105 mm Hg throughout); 835 patients (43.2%) as moderate-declining, with intermediate MAP levels (93.3 ± 13.4 mm Hg at baseline, declining to approximately 86–88 mm Hg); and 687 patients (35.5%) as low-persistent, exhibiting persistently low MAP values (80.0 ± 14.6 mm Hg at baseline, stabilizing around 72–74 mm Hg). External validation in the eICU cohort demonstrated similar distribution patterns, with 826 (29.5%), 1128 (40.3%), and 846 (30.2%) patients assigned to High-Stable, Moderate-Declining, and Low-Persistent subtypes, respectively.

### 3.4. Survival analysis by MAP trajectory subtypes

**Figure 4.**
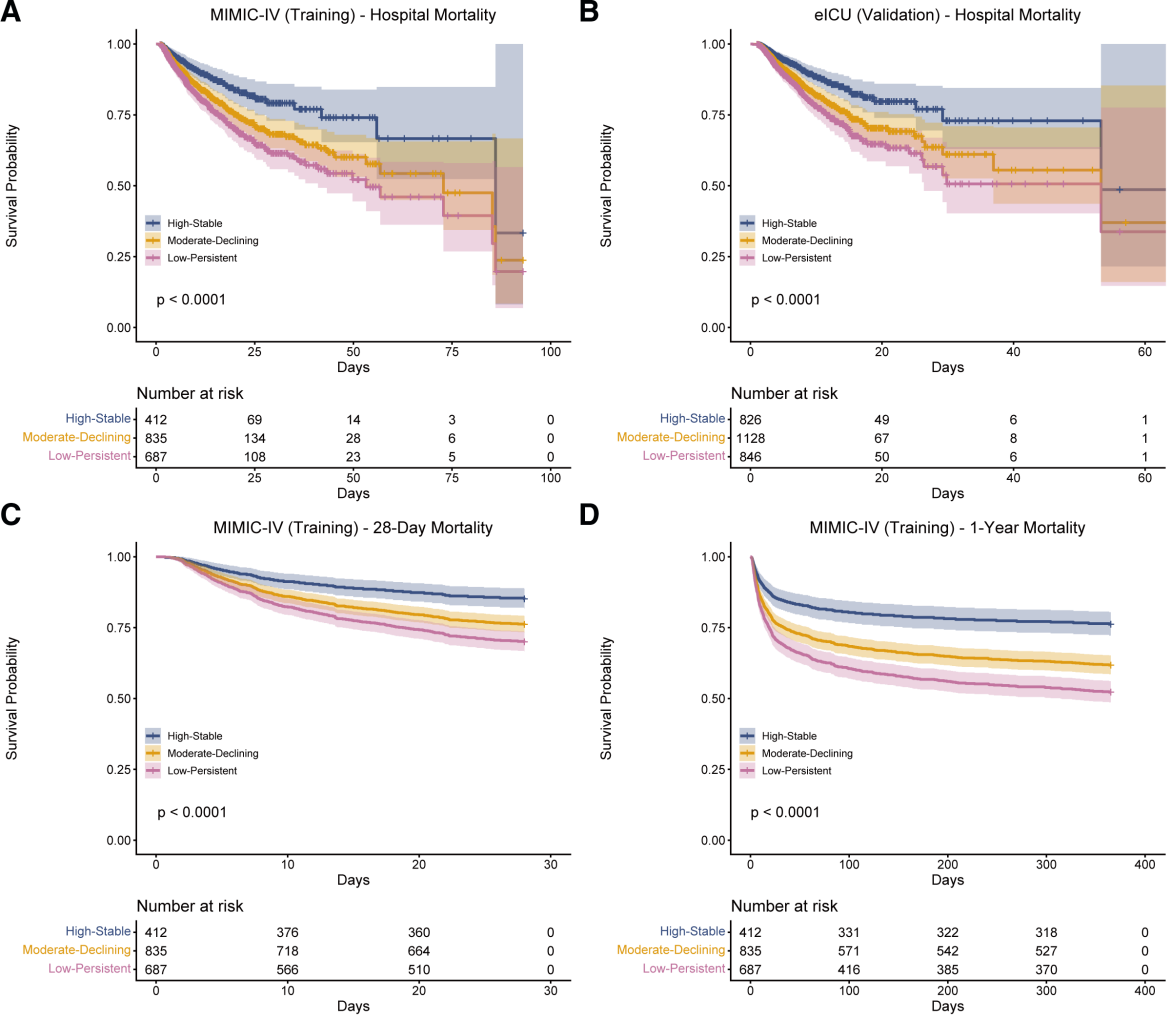
Kaplan–Meier survival curves comparing mortality outcomes among MAP trajectory subtypes. The High-Stable subtype (blue) demonstrates the most favorable survival, followed by Moderate-Declining (orange) and Low-Persistent (pink). *P* values are derived from log-rank tests. (A) In-hospital mortality in MIMIC-IV training cohort; (B) In-hospital mortality in eICU validation cohort; (C) 28-day mortality in MIMIC-IV cohort; (D) 1-year mortality in MIMIC-IV cohort. ICU = intensive care unit, MAP = mean arterial pressure, MIMIC-IV = Medical Information Mart for Intensive Care IV.

Kaplan–Meier survival analysis demonstrated significant differences in mortality outcomes among the 3 trajectory subtypes across all endpoints (Fig. 4). In the MIMIC-IV training cohort, the High-Stable subtype exhibited the lowest in-hospital mortality rate (11.9%, 49/412), followed by the Moderate-Declining subtype (18.9%, 158/835), and the Low-Persistent subtype (23.6%, 162/687; log-rank *P* < .0001; Fig. 4A). Cox proportional hazards regression confirmed these associations, with the Moderate-Declining subtype showing a significantly increased risk of in-hospital mortality compared to the High-Stable reference group (HR: 1.62, 95% CI: 1.17–2.23, *P* = .003), and the Low-Persistent subtype demonstrating the highest risk (HR: 2.03, 95% CI: 1.47–2.79, *P* < .001).

The prognostic value of MAP trajectory subtypes was consistent across secondary outcomes in the MIMIC-IV cohort. For 28-day mortality (Fig. 4C), event rates increased progressively from High-Stable (14.8%) to Moderate-Declining (23.8%) to Low-Persistent (30.0%; *P* < .0001), with corresponding hazard ratios of 1.70 (95% CI: 1.27–2.26) and 2.22 (95% CI: 1.67–2.95), respectively. Similar gradients were observed for 1-year mortality (Fig. 4D), with rates of 23.8%, 38.2%, and 47.7% across subtypes (*P* < .0001), and hazard ratios of 1.76 (95% CI: 1.41–2.21) and 2.36 (95% CI: 1.88–2.96). Importantly, external validation in the eICU cohort (Fig. 4B) confirmed these findings, demonstrating in-hospital mortality rates of 8.2%, 13.1%, and 16.4% for High-Stable, Moderate-Declining, and Low-Persistent subtypes, respectively (*P* < .0001), with comparable hazard ratios of 1.61 (95% CI: 1.21–2.14) and 2.01 (95% CI: 1.51–2.69).

### 3.5. Clinical characteristics by MAP trajectory subtypes

**Figure 5.**
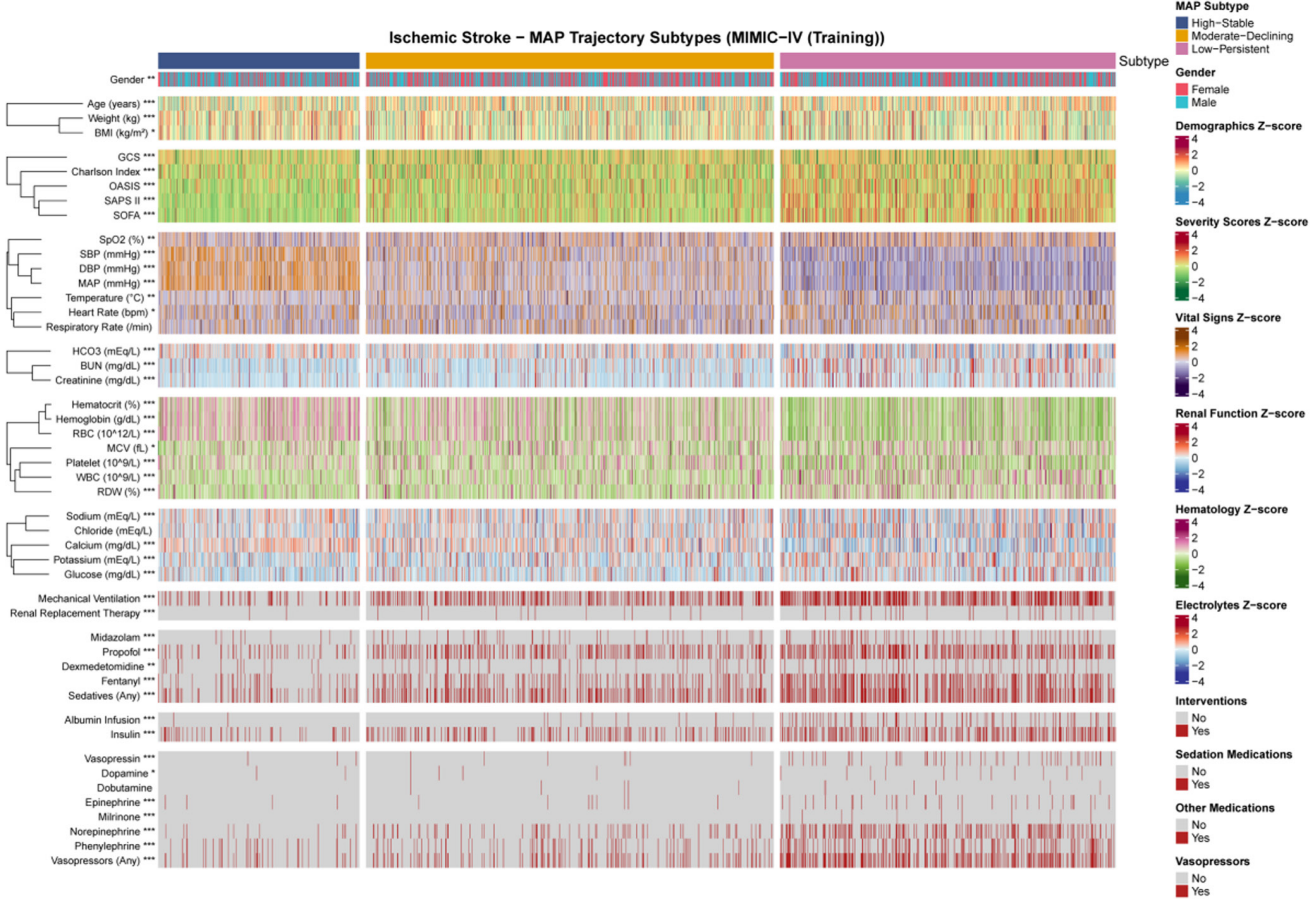
Heatmap visualization of baseline clinical features across MAP trajectory subtypes in the MIMIC-IV training cohort. Continuous variables are displayed as standardized *z*-scores, and categorical variables as proportions. Asterisks indicate statistical significance: *P* < .05, *P* < .01, *P* < .001. Trajectory subtypes: 1 = High-Stable, 2 = Moderate-Declining, 3 = Low-Persistent. MAP = mean arterial pressure, MIMIC-IV = Medical Information Mart for Intensive Care IV.

To explore the clinical profiles underlying the identified trajectory subtypes, baseline characteristics were compared across the 3 groups in the MIMIC-IV training cohort (Fig. 5; Table S4, Supplemental Digital Content). Patients in the Low-Persistent subtype were significantly older (70.50 ± 14.87 years) compared to the Moderate-Declining (68.88 ± 15.35 years), and High-Stable subtypes (67.05 ± 14.33 years; *P* < .001). Disease severity scores demonstrated a clear gradient across subtypes, with the Low-Persistent group exhibiting the highest Simplified Acute Physiology Score II (42.39 ± 14.51 vs 34.71 ± 12.43 vs 29.46 ± 10.11), OASIS (35.08 ± 8.84 vs 31.94 ± 8.26 vs 30.00 ± 7.34), SOFA (5.91 ± 3.69 vs 3.81 ± 2.99 vs 2.54 ± 2.27), and Charlson comorbidity index (7.44 ± 2.89 vs 6.63 ± 2.93 vs 6.43 ± 2.58; all *P* < .001).

Regarding laboratory parameters, patients in the Low-Persistent subtype demonstrated worse renal function with higher creatinine (1.52 ± 1.23 vs 1.21 ± 1.01 vs 1.12 ± 0.81 mg/dL) and blood urea nitrogen levels (29.01 ± 20.99 vs 21.59 ± 15.18 vs 19.09 ± 13.51 mg/dL), as well as more pronounced anemia with lower hemoglobin (10.43 ± 2.29 vs 11.64 ± 2.30 vs 12.49 ± 2.24 g/dL; all *P* < .001). Furthermore, the Low-Persistent subtype required significantly more intensive interventions, including higher rates of mechanical ventilation (49.5% vs 32.0% vs 15.8%), vasopressor use (40.2% vs 16.6% vs 9.7%), RRT (4.8% vs 1.7% vs 0.5%), and sedative administration (43.7% vs 31.3% vs 16.3%; all *P* < .001). Similar patterns were observed in the eICU external validation cohort (Fig. S1, Supplemental Digital Content).

### 3.6. Subgroup analyses

**Figure 6.**
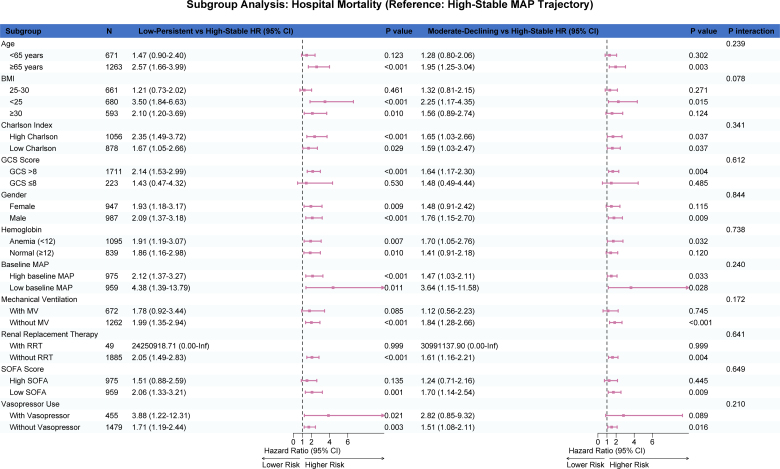
Forest plot depicting categorized exploratory subgroup analyses for the association between MAP trajectory subtypes and in-hospital mortality. Hazard ratios with 95% confidence intervals are shown for Low-Persistent versus High-Stable and Moderate-Declining versus High-Stable comparisons across clinically relevant subgroups. These categorized analyses are presented for descriptive and clinical interpretability purposes. Continuous interaction analyses for major continuous covariates are provided in Figures S4,S5 and S6, Supplemental Digital Content as the primary assessment of potential effect modification. MAP = mean arterial pressure.

Exploratory effect-modification analyses were conducted to examine whether the association between MAP trajectory subtypes and mortality varied across prespecified clinical variables. In the primary heterogeneity analyses, continuous interaction models were fitted for age, BMI, SOFA score, GCS score, Charlson comorbidity index, baseline MAP, and hemoglobin (Figs. S4,S5 and S6, Supplemental Digital Content). Overall, these continuous interaction analyses showed that the direction of association for the Low-Persistent and Moderate-Declining subtypes relative to the High-Stable subtype was broadly preserved across the observed range of these covariates, without an apparent qualitative change in the main findings. For complementary descriptive presentation, categorized subgroup analyses are shown in Figure 6 and Figures S2 and S3, Supplemental Digital Content. These forest plots similarly demonstrated a broadly consistent direction of association across most strata. Because the categorized analyses were exploratory and involved clinically simplified or sample-dependent cutoffs, they should be interpreted as descriptive complements to the continuous interaction models rather than as the primary basis for inference regarding effect heterogeneity.

## 4. Discussion

We identified 3 distinct MAP trajectory subtypes during the first 24 hours after ICU admission in patients with ischemic stroke and observed a graded association with mortality. In the MIMIC-IV cohort, compared with the High-Stable group, the Moderate-Declining trajectory was associated with higher in-hospital mortality (HR 1.62, 95% CI 1.17–2.23), whereas the Low-Persistent trajectory conferred the highest risk (HR 2.03, 95% CI 1.47–2.79). Similar gradients were observed for 28-day and 1-year mortality in MIMIC-IV and for in-hospital mortality in the external eICU cohort. Collectively, these findings suggest that early post-ICU admission MAP trajectories capture heterogeneous hemodynamic patterns associated with adverse outcomes. However, the present study did not directly compare trajectory-based measures with static MAP indices and therefore cannot determine whether trajectory subtypes provide incremental prognostic value beyond conventional single-time-point or summary MAP measures.

Our findings complement a growing body of stroke literature suggesting that temporal blood pressure patterns, including trajectories and variability, may provide information complementary to static measurements. Contemporary studies have demonstrated distinct systolic blood pressure trajectory groups during the first 24 to 72 hours after EVT and reported outcome differences across trajectory phenotypes.^[17,20]^ In addition, higher blood pressure variability during the first 24 hours after successful thrombectomy has been associated with worse outcomes, including in basilar artery occlusion,^[15]^ and increased beat-to-beat blood pressure variability within 24 hours of ischemic stroke onset has been linked to unfavorable 90-day prognosis.^[13]^ While most prior work has focused on systolic blood pressure and post-reperfusion settings, our study extends the dynamic BP phenotype concept to MAP and specifically targets the ICU admission window, a period characterized by intensive stabilization, fluctuating vasopressor and sedation exposure, and high susceptibility to systemic complications.

Several pathophysiological considerations may explain why a persistently low MAP trajectory during early ICU care is associated with worse outcomes. Cerebral autoregulation is frequently impaired after ischemic stroke, increasing dependence of cerebral blood flow on systemic perfusion pressure and potentially rendering the injured brain more vulnerable to sustained or recurrent hypotension.^[21,22]^ In parallel, hypotension is a heterogeneous clinical state that may reflect impaired cardiac output, vasoplegia, sedation-related vasodilation, bleeding, or sepsis physiology, each with distinct implications for end-organ perfusion and outcomes.^[23]^ Evidence from acute reperfusion contexts further supports the potential harm of hypotension magnitude; for example, greater intra-procedural hypotension magnitude during thrombectomy under general anesthesia has been associated with poor neurological outcome.^[16]^ Although our analysis centers on the post-ICU admission phase rather than intra-procedural hemodynamics, the directionality of these data is consistent with the observation that sustained low MAP identifies a clinically high-risk phenotype.

The clinical profiles of the trajectory groups also support the interpretation that early MAP dynamics integrate both neurologic vulnerability and systemic illness burden. Patients in the Low-Persistent group exhibited higher acuity and greater early use of organ support and vasoactive therapies, suggesting that persistent hypotension may represent a composite signal of severe physiological derangement and treatment intensity. Notably, the mortality associations persisted after multivariable adjustment, arguing that trajectory membership is not merely a surrogate of baseline severity, although residual confounding remains possible. This is particularly relevant in ICU cohorts, where treatment decisions (e.g., vasopressor titration, sedation depth, fluid strategy) are tightly coupled to evolving physiology and may introduce confounding by indication.

To better assess potential effect modification, we complemented the categorized subgroup analyses with continuous interaction models for several key continuous covariates, including age, BMI, SOFA score, GCS score, Charlson comorbidity index, baseline MAP, and hemoglobin. These analyses were undertaken to avoid overreliance on arbitrary or sample-dependent cutoffs and to preserve more of the underlying information in the data. Overall, the continuous interaction analyses yielded patterns broadly consistent with the categorized forest plots, supporting the general robustness of the association between less favorable MAP trajectory subtypes and mortality across a range of clinical profiles. Nevertheless, these analyses remained exploratory and should not be interpreted as definitive evidence for the absence of effect heterogeneity.

From a practical standpoint, our findings suggest that early MAP behavior over the first ICU day may reflect clinically relevant hemodynamic phenotypes associated with prognosis in critically ill patients with ischemic stroke. Nevertheless, these results should be interpreted cautiously. Continuous hemodynamic assessment is already part of routine ICU care, and our study does not establish that trajectory-based classification is superior to conventional bedside assessment or static MAP measures. Therefore, the present findings are better viewed as hypothesis-generating and as supportive of further investigation into whether dynamic MAP phenotyping may complement, rather than replace, existing clinical assessment frameworks.^[10–12]^ Observational evidence likewise suggests that medication-induced BP decreases within the first 24 hours after successful recanalization may be associated with poorer outcomes.^[24]^

Future research should directly compare trajectory-based MAP phenotypes with conventional static MAP metrics, and should evaluate whether integrating MAP trajectories with treatment intensity, neurologic severity, imaging findings, and biochemical markers can improve risk stratification or guide individualized hemodynamic strategies. Prospective studies in contemporary neurocritical care cohorts will be particularly important to determine the clinical utility, reproducibility, and bedside applicability of these trajectory patterns^[6]^

### 4.1. Strengths and limitations

Strengths of this study include the trajectory-based characterization of early MAP behavior during a clinically relevant window, namely the first 24 hours after ICU admission, and the use of 2 large critical care databases with external validation. These features support the reproducibility of the identified trajectory patterns and of their associations with mortality. In addition, the analysis incorporated a broad set of demographic, clinical, laboratory, and treatment-related covariates, which improved adjustment for measured differences in patient acuity and management.

Several limitations should be acknowledged. First, this was an observational study and cannot establish causality; residual confounding and confounding by indication remain possible. Second, the study did not directly compare trajectory subtypes with conventional static MAP measures, such as admission MAP or summary MAP indices, and therefore cannot determine whether trajectory-based classification provides incremental prognostic information beyond standard hemodynamic assessment. Third, trajectory modeling required complete hourly MAP data across the first 24 hours after ICU admission; therefore, selection bias related to data completeness cannot be excluded, and generalizability may be limited for patients with shorter stays or incomplete early hemodynamic monitoring. Fourth, several treatment variables were captured within the same early ICU window used to define MAP trajectories. As a result, adjustment for these variables may not fully separate baseline confounding from treatment-response pathways. Fifth, key stroke-specific variables, including infarct volume, occlusion site, reperfusion status, and detailed neurologic severity measures, were unavailable or incomplete in these ICU databases, limiting mechanistic inference and preventing assessment of functional outcomes beyond mortality. Sixth, analyses of effect modification were exploratory. Although we supplemented the categorized subgroup displays with continuous interaction models for major continuous covariates, these assessments were not prespecified as primary analyses and may still have been underpowered to detect modest heterogeneity. Therefore, the absence of clear interaction in the present study should not be interpreted as conclusive evidence of homogeneous effects across all clinical subgroups. Finally, trajectory classification is model-dependent, and alternative modeling choices could yield different group structures; prospective validation in dedicated neurocritical care cohorts remains necessary.

## Acknowledgments

The authors acknowledge the researchers and institutions responsible for the MIMIC-IV and eICU databases for making these valuable resources publicly available.

## Author contributions

**Data curation:** Jintao Qiu.

**Formal analysis:** Jintao Qiu.

**Investigation:** Jintao Qiu.

**Project administration:** Jintao Qiu.

**Resources:** Jintao Qiu.

**Software:** Jintao Qiu, Rongrong Liu.

**Supervision:** Rongrong Liu.

**Validation:** Jintao Qiu.

**Visualization:** Jintao Qiu, Rongrong Liu.

**Writing – original draft:** Jintao Qiu, Rongrong Liu.

**Writing – review & editing:** Jintao Qiu, Rongrong Liu.









**Figure:**
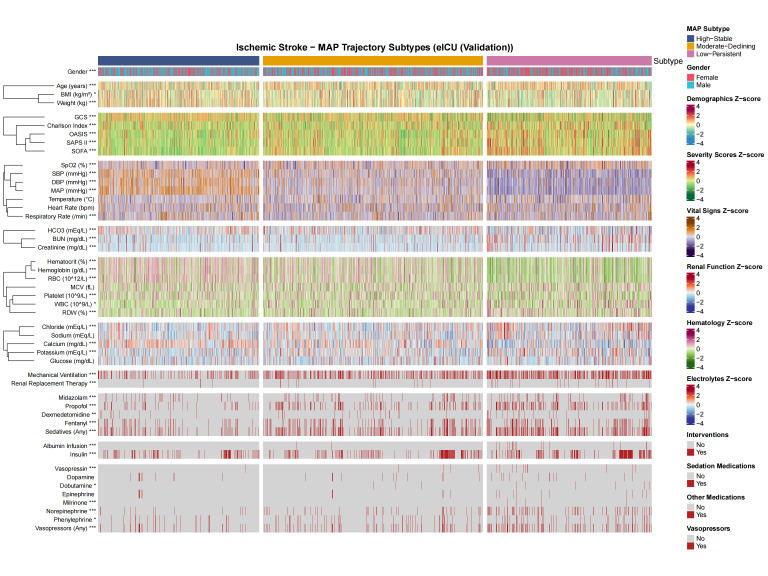


**Figure:**
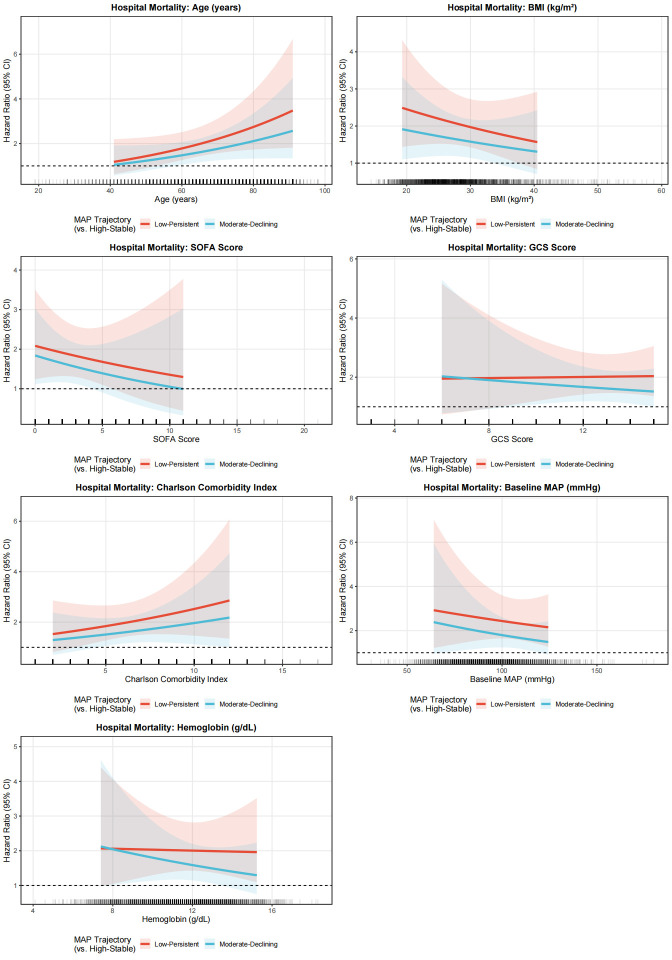


**Figure:**
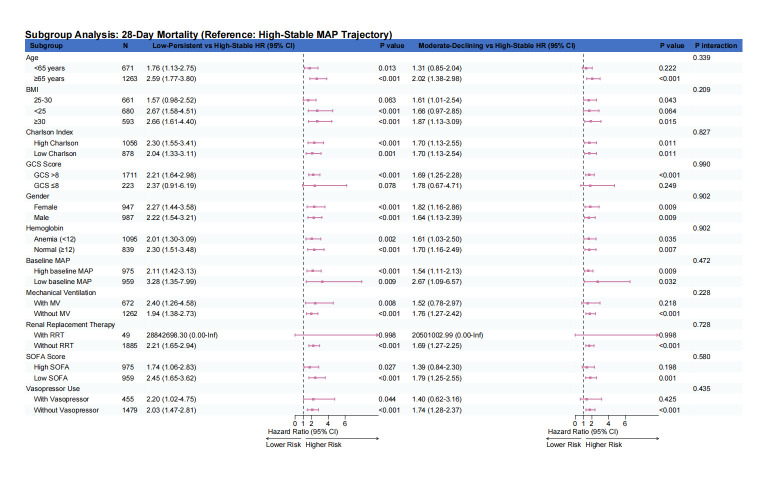


**Figure:**
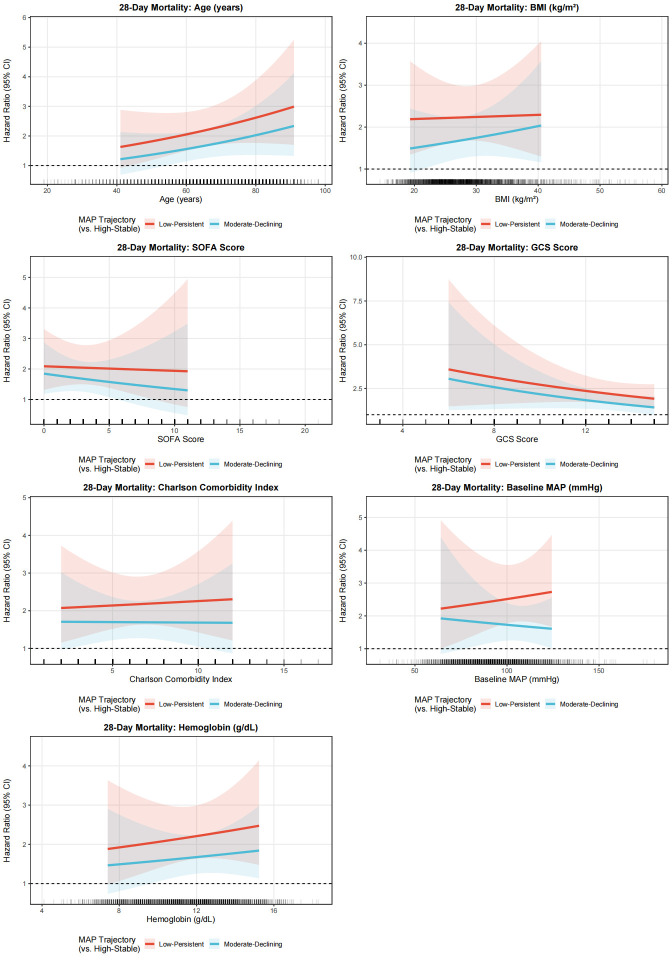


**Figure:**
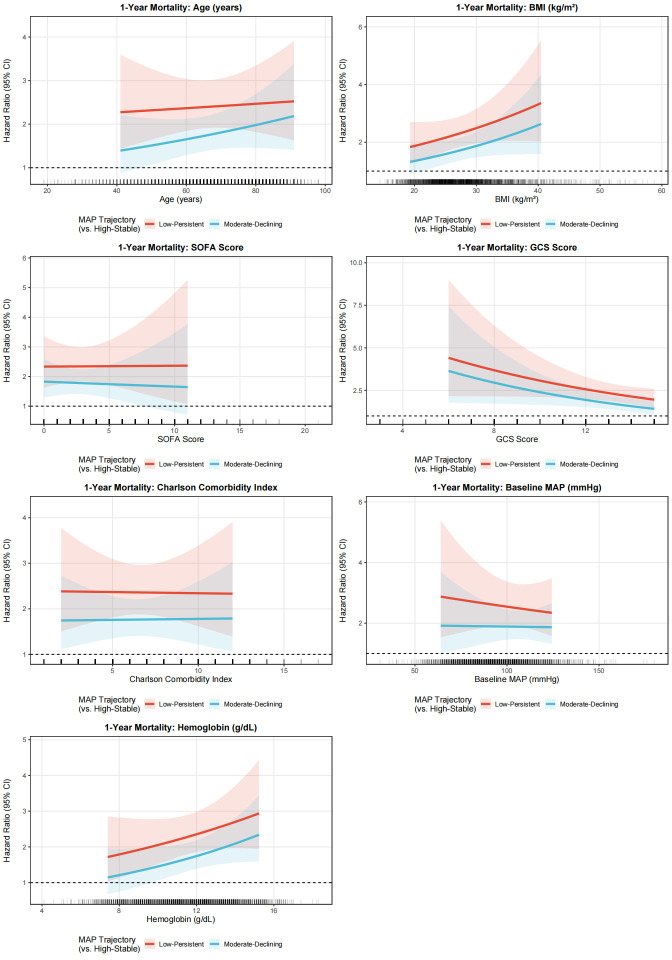


**Figure:**